# Exploring eating behaviors, knowledge and attitudes of adolescent Indian girls

**DOI:** 10.6026/973206300200165

**Published:** 2024-02-29

**Authors:** N Sivasubramanian, Desai Nehaben Jashubhai, B Mahalakshmi, Gnanadesigan Ekambaram, Ravi kant

**Affiliations:** 1 Department of Psychiatric Nursing, Nootan College of Nursing, Sankalchand Patel University, Visnagar, Gujarat - 384315, India; 2 Department of Paediatric Nursing, Nootan College of Nursing, Sankalchand Patel University, Visnagar, Gujarat - 384315; India; 3 Department of Physiology, Nootan Medical College & Research Centre, Sankalchand Patel University, Visnagar, Gujarat, India; 4 Department of Microbiology, Nootan Medical College & Research Centre, Sankalchand Patel University, Visnagar, Gujarat, India

**Keywords:** Adolescent health, eating behaviors, knowledge, attitudes, non-communicable diseases

## Abstract

Data on the eating behaviors, knowledge, and attitudes of adolescent girls in Visnagar, India, focusing on the prevalence of non-communicable diseases [NCDs] and their association with dietary practices is of interest. Adolescence, a crucial developmental phase, sets the foundation for lifelong health habits, necessitating an understanding of the determinants influencing eating behaviors. The research aims to identify gaps in knowledge, attitudes, and practices [KAP], providing insights for culturally sensitive public health strategies. Through structured questionnaires and Likert scales, data were collected from a purposive sample of adolescent girls [ages 12-18] in a selected school. Descriptive statistics and correlation analyses were employed to assess knowledge, attitudes, and behaviors, considering demographic variables. Non-significant associations were found between these variables and demographics. The mean knowledge score was 25.11, reflecting a moderate level, while the mean attitudes score was 99.54, indicating generally positive attitudes. Adolescent girls demonstrated an overall mean behaviour score of 110.93, with a positive correlation [0.72] between knowledge and behaviors and a stronger correlation [0.99] between attitudes and behaviors. Findings highlight the universal importance of knowledge in influencing eating behaviors and emphasize the need for culturally tailored interventions considering regional influences. The study contributes valuable insights into the interplay of knowledge, attitudes, and behaviors related to eating disorders in adolescent girls, serving as a foundation for targeted public health strategies.

## Background:

In recent years, the surge in non-communicable diseases [NCDs] has evolved into a pressing global public health crisis. Data from the World Health Organization [WHO] reveals that an estimated 71% of all global deaths are attributed to NCDs, with diet-related factors being major contributors [1]. According to the Global Burden of Disease Study 2019, cardiovascular diseases stand out as the leading cause of mortality worldwide, causing approximately 17.9 million deaths annually [2]. As we grapple with this significant public health concern, evidence-backed initiatives, informed by robust data, are essential. By understanding the quantitative dimensions of the NCD epidemic, policymakers, healthcare professionals, and public health advocates can formulate targeted interventions that address the specific challenges posed by diet-related factors [3]. In recent years, the global rise in non-communicable diseases [NCDs] has become a major public health concern, with a substantial portion of adult deaths attributed to diet-related NCDs, including cardiovascular diseases, cancers, and diabetes mellitus. [4] Adolescence represents a critical period of development characterized by numerous physical, psychological, and social changes, including the establishment of lifelong health behaviour [5]. As adolescence is a formative period for lifelong habits, uncovering the determinants of eating behaviors in this context holds profound implications for public health interventions and targeted educational initiatives [6]. Adolescents, a pivotal demographic in the development of lifelong habits, are particularly vulnerable to unhealthy dietary practices, as evidenced by the growing prevalence of adolescent obesity [7]. Understanding the intricate interplay of knowledge, attitudes, and practices [KAP] among adolescents is crucial for devising effective interventions to address the escalating risks associated with NCDs[8].The existing literature underscores the significance of KAP elements in driving behavioural change, with an emphasis on their role in shaping personal eating habits. While foundational nutritional knowledge is typically imparted through school curricula, there remains a dearth of comprehensive exploration into various facets of food knowledge among adolescent [9]. Adolescent dietary choices are influenced by a multitude of factors, ranging from taste preferences and cost considerations to broader socio-cultural norms. Despite acknowledging the importance of healthy eating, adolescents often grapple with conflicting factors that impact their food choices, such as flavour, cost, and convenience [10]. Moreover, the prevailing eating culture and cooking methods in Visnagar, India, present a unique context that requires closer examination. Therefore, it is of interest to bridge existing gaps in the understanding of adolescent eating behaviors by conducting a descriptive exploration in Visnagar, India.

## Methodology:

## Study Design:

This research employed a descriptive study design to comprehensively assess the eating behaviors, knowledge, and attitudes of adolescent girls regarding eating disorders in a selected school in Visnagar.

## Study Setting:

The study was conducted in a specific school in Visnagar, providing a focused environment for the assessment of eating behaviors among adolescent girls.

## Sampling:

A purposive sampling method was utilized to select the study participants [11]. Adolescent girls within the age range of 12-18 years from the selected school were included in the study.

## Data collection:

## Structured questionnaires:

The study employed structured questionnaires to gather data on eating behaviors, knowledge of eating disorders, and attitudes towards such disorders. The questionnaires were designed to capture relevant information while ensuring participant comfort and understanding.

## Likert Scales:

Likert scales were utilized to assess the attitudes of adolescent girls towards eating disorders. The scales provided a quantifiable measure of attitudes, allowing for a nuanced understanding of participants' perspectives.

## Data analysis:

Descriptive statistics, including mean scores and standard deviations, were calculated to analyze the overall eating behaviors, knowledge, and attitudes of adolescent girls. Correlation analyses were conducted to explore relationships between eating behaviour, knowledge, and attitudes. Statistical significance was determined at a p-value of 0.05.

## Ethical considerations:

The study adhered to ethical principles, ensuring the confidentiality and anonymity of participants. Informed consent was obtained from both participants and their guardians, emphasizing the voluntary nature of participation.

## Results:

**Figure 1:**
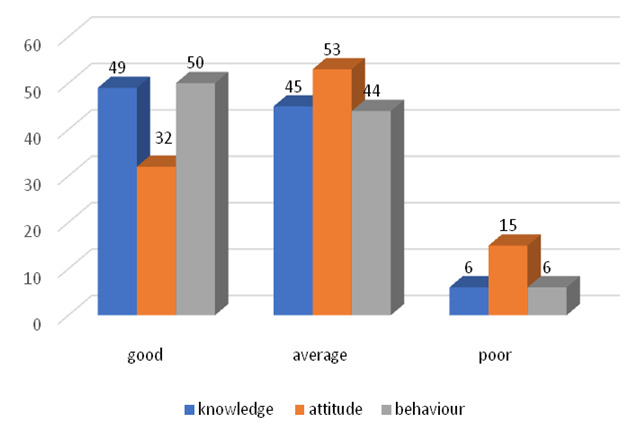
Distribution of sample as per their knowledge, attitude and behaviour category

Table 1 show the age distribution reveals that 33 participants are between 12-14 years old, while 67 fall within the 14-18 age range. Height is categorized into four groups, with the majority of participants [48] falling between 151 cm to 155 cm. Weight distribution shows that 48 participants weigh between 41 kg to 45 kg, and the majority follow a vegetarian diet [95 individuals]. Table 2 presents the chi-square values computed between the level of knowledge, eating attitude, eating behaviour, and selected demographic variables. The analysis indicates non-significant associations across all demographic variables with the levels of knowledge, eating attitude, and eating behaviour. Specifically, the chi-square values for age, height, weight, religion, place of residence, type of family, income, dietary habits, mother's occupation, and father's occupation are reported, and all of them are non-significant (Figure 1).

The overall mean knowledge score was 25.11 with a standard deviation of 2.22, while the mean eating attitudes score was 99.54 with a standard deviation of 6.32. The adolescent girls demonstrated an overall mean eating behaviour score of 110.93 with a standard deviation of 16.30. Correlation analyses revealed a positive association [0.72] between eating behaviour and knowledge, suggesting that higher knowledge is linked to better eating behaviors. Furthermore, a stronger positive correlation [0.99] was observed between eating behaviour and eating attitudes, signifying a significant connection. This indicates that improved eating attitudes are associated with healthier eating behaviors among the studied population. The study contributes valuable insights into the interplay of knowledge, attitudes, and behaviors related to eating disorders in adolescent girls.

## Discussion:

The study's focus on eating attitudes and behaviors among adolescent girls is particularly relevant given the unique challenges this demographic faces, including societal expectations, body image concerns, and an increasing prevalence of disordered eating behaviors. The findings provide insights into the complex interplay of these factors and their implications for public health. To contextualize and enrich our understanding, we compare the results of the current study with findings from three relevant studies in each area. Culturally tailored interventions should prioritize addressing these specific challenges to foster positive eating attitudes and behaviors.

However, a study by Liu *et al.* [2021] in an Asian population found a higher level of knowledge, suggesting cultural variations in nutritional awareness among adolescents [12]. The positive correlation [0.72] observed in the present study between knowledge and eating behaviors is consistent with the findings of Almansour *et al.* [2020], who highlighted the role of nutritional knowledge in shaping healthier dietary practices among adolescents [13]. These consistent correlations emphasize the universal importance of knowledge in influencing eating behaviors.

The study revealed a moderate level of nutritional knowledge among adolescent girls in Visnagar, as reflected in the mean knowledge score of 25.11. This finding aligns with studies conducted in both Western and Asian populations, emphasizing a consistent moderate level of knowledge among adolescents globally [14]. : The positive correlation [0.72] observed between knowledge and eating behaviors as shown elsewhere [15], emphasizing the universal importance of knowledge in influencing healthier dietary practices [16].

The moderate level of knowledge underscores the need for targeted education interventions to enhance nutritional awareness among adolescent girls in Visnagar. Context-specific educational initiatives should address cultural nuances and dietary practices prevalent in the region. Public health campaigns, school-based programs, and community workshops could play pivotal roles in disseminating accurate and culturally sensitive nutritional information.

The study revealed generally positive eating attitudes among adolescent girls in Visnagar, with a mean attitude score of 99.54. The strong correlation [0.99] between eating attitudes and behaviors highlights the significance of cultivating positive attitudes for promoting healthier dietary practices. This finding is consistent with research by Aggarwal *et al.* 2020 [17]. The strong correlation suggests that interventions focusing on improving attitudes may effectively translate into healthier eating behaviors among this population. In contrast, a study by Yang *et al.* [2010] reported a weaker correlation between attitudes and behaviors among Korean adolescents, highlighting potential cultural nuances [18]. The variance in these correlations underscores the need for culturally tailored interventions that consider regional influences on eating attitudes and behaviors. The non-significant associations between knowledge, attitudes, and behaviors with demographic variables highlight the need for universal interventions that transcend socio-demographic differences. These findings align with research by Rachel Brown, *et al.* [2018], suggesting that nutritional knowledge and behaviors among adolescents may be minimally influenced by demographic factors [19, 20]. However, it is crucial to note that this contrasts with the findings of Lirong *et al.* [2023], where significant associations were identified between dietary behaviors and certain demographic factors [21].

This discrepancy underscores the importance of considering context-specific factors when designing interventions. The study's insights provide a foundation for developing context-specific public health strategies in the on-going battle against diet-related NCDs. Universal interventions that promote healthier dietary practices, foster nutritional knowledge, and cultivate positive attitudes toward food are warranted. However, these interventions should be sensitive to regional influences, cultural norms, and the specific challenges faced by adolescent girls in Visnagar. Given the positive correlation between knowledge and behaviors, educational interventions should be prioritized to enhance nutritional knowledge. Schools, as key settings for adolescent development, can serve as effective platforms for implementing targeted educational programs. Integrating nutrition education into the school curriculum and organizing awareness campaigns can empower adolescents with the knowledge needed to make informed dietary choices. Interventions should focus on promoting positive eating attitudes, recognizing their strong correlation with actual behaviors. Culturally sensitive initiatives that address societal expectations, body image concerns, and the unique cultural context of Visnagar are essential. Involving parents, teachers, and community leaders in these initiatives can create a comprehensive support system for adolescents.

## Conclusion:

Data gives insights into the eating behaviors, knowledge, and attitudes of adolescent girls in Visnagar, India. The findings emphasize the need for targeted education initiatives to enhance nutritional knowledge, promote positive eating attitudes, and foster healthier dietary practices. Context-specific interventions that consider regional influences and address the unique challenges faced by adolescent girls are crucial for the development of effective public health strategies. Ultimately, fostering informed choices and positive attitudes during adolescence holds the key to establishing lifelong healthy habits and mitigating the impact of diet-related NCDs.

## Figures and Tables

**Table 1:** Demographic Characteristics of the Sample

**S No.**	**Variables**	**Classification**	**Frequency**
1	Age in years	Dec-14	33
		14-18	67
2	Height	Below 150 cm	6
		151 cm to 155 cm	48
		156 cm to 160 cm	42
		161 cm to 165 cm	4
3	Weight	Below 40 kg	5
		41 kg to 45 kg	48
		46 kg to 50 kg	44
		51 kg to 55 kg	3
4	Religion	Hindu	93
		Muslim	5
		Christian	2
5	Place of residence	Urban	49
		Rural	49
		Slum	2
6	Type of family	Nuclear	21
		Joint	79
7	Income	<12,000	23
		12,000-15,000	24
		15,000-18,000	33
		> 18,000	20
8	Dietary habit	Vegetarian	95
		Non-vegetarian	5
9	Mother's occupation	Housewife	62
		Employee	22
		Agricultural laborer	12
		Non-agricultural laborer	4
10	Father's occupation	Business	55
		Employee	36
		Agricultural laborer	7
		Non-agricultural laborer	7

**Table 2:** Chi-Square Values Computed between Level of Knowledge, Eating Attitude, Eating Behaviour, and Selected Demographic Variables

**Demographic Variables**	**Level of Knowledge**	**Eating Attitude**	**Eating Behavior**
Age	0.117 [Non Sig]	0.77 [Non Sig]	0.397 [Non Sig]
Height	2.256 [Non Sig]	1.849 [Non Sig]	7.101 [Non Sig]
Weight	0.797 [Non Sig]	0.568 [Non Sig]	5.372 [Non Sig]
Religion	2.569 [Non Sig]	0.48 [Non Sig]	2.12 [Non Sig]
Place of Residence	0.322 [Non Sig]	0.854 [Non Sig]	3.031 [Non Sig]
Type of Family	0.003 [Non Sig]	0.072 [Non Sig]	2.082 [Non Sig]
Income	6.842 [Non Sig]	7.587 [Non Sig]	3.959 [Non Sig]
Dietary Habits	2.493 [Non Sig]	0.336 [Non Sig]	0.361 [Non Sig]
Mother's Occupation	5.922 [Non Sig]	2.907 [Non Sig]	3.508 [Non Sig]
Father's Occupation	4.306 [Non Sig]	0.951 [Non Sig]	2.869 [Non Sig]
Non Sig: Non-Significant
